# Distribution and Niche Separation of Planktonic Microbial Communities in the Water Columns from the Surface to the Hadal Waters of the Japan Trench under the Eutrophic Ocean

**DOI:** 10.3389/fmicb.2016.01261

**Published:** 2016-08-10

**Authors:** Takuro Nunoura, Miho Hirai, Yukari Yoshida-Takashima, Manabu Nishizawa, Shinsuke Kawagucci, Taichi Yokokawa, Junichi Miyazaki, Osamu Koide, Hiroko Makita, Yoshihiro Takaki, Michinari Sunamura, Ken Takai

**Affiliations:** ^1^Marine Functional Biology Group, Research and Development Center for Marine Biosciences, Japan Agency for Marine-Earth Science and TechnologyYokosuka, Japan; ^2^Department of Subsurface Geobiological Analysis and Research, Japan Agency for Marine-Earth Science and TechnologyYokosuka, Japan; ^3^Department of Earth and Planetary Science, The University of TokyoTokyo, Japan

**Keywords:** hadal, trench, nitrification, deep ocean, niche separation

## Abstract

The Japan Trench is located under the eutrophic Northwestern Pacific while the Mariana Trench that harbors the unique hadal planktonic biosphere is located under the oligotrophic Pacific. Water samples from the sea surface to just above the seafloor at a total of 11 stations including a trench axis station, were investigated several months after the Tohoku Earthquake in March 2011. High turbidity zones in deep waters were observed at most of the sampling stations. The small subunit (SSU) rRNA gene community structures in the hadal waters (water depths below 6000 m) at the trench axis station were distinct from those in the overlying meso-, bathy and abyssopelagic waters (water depths between 200 and 1000 m, 1000 and 4000 m, and 4000 and 6000 m, respectively), although the SSU rRNA gene sequences suggested that potential heterotrophic bacteria dominated in all of the waters. Potential niche separation of nitrifiers, including ammonia-oxidizing archaea (AOA), was revealed by quantitative PCR analyses. It seems likely that *Nitrosopumilus*-like AOAs respond to a high flux of electron donors and dominate in several zones of water columns including shallow and very deep waters. This study highlights the effects of suspended organic matter, as induced by seafloor deformation, on microbial communities in deep waters and confirm the occurrence of the distinctive hadal biosphere in global trench environments hypothesized in the previous study.

## Introduction

Hadal oceans at water depths of below 6000 m are composed almost exclusively of trenches and are one of the least-explored biospheres on Earth. Trench environments are hydro topographically isolated from upper oceans, and exhibit elevated hydrostatic pressure, whereas the other physical and chemical conditions, such as temperature, salinity, dissolved oxygen (DO), and nutrient concentrations, in the hadal waters are comparable to those in abyssal oceans at a water depth of between 4000 and 6000 m (Mantyla and Reid, 1978; Nozaki et al., 1998; Taira et al., 2004, 2005; Jamieson et al., 2010). Currently the existence of the “hadal biosphere” has been demonstrated for both benthic and planktonic microbial ecosystems in the Challenger Deep of the Mariana Trench (Glud et al., 2013; Nunoura et al., 2015). The hadal benthic microbial ecosystems in the Mariana Trench have been characterized by their higher carbon turnover and oxygen consumption rates than those in adjacent abyssal plain sediments (Glud et al., 2013). Planktonic hadal microbial communities harbor relatively higher abundances of heterotrophic bacterial populations and nitrifiers populations probably adapting higher fluxes of electron donors as compared with the communities associated with the overlying abyssal water masses (Nunoura et al., 2015). The hadal biosphere and biogeochemical cycles are likely constrained primarily by the trench geomorphology and by the endogenous recycling and input of organic matter associated with occasional seafloor deformation (landslides and turbulent sediments) in the vast trench slope area (Glud et al., 2013; Ichino et al., 2015; Nunoura et al., 2015). Nevertheless, such a distinctive hadal microbial ecosystem has been found only in the Mariana Trench, and the following points should be addressed further: whether the distinctive hadal microbial ecosystem is widespread in the global trench environments; whether the members and functions of hadal microbial communities are common in trenches under geologically and oceanographically different settings or specific in each hadal environment; and if different, what types of factors constrain the compositions and functions of hadal microbial communities and how they are controlled.

The Japan Trench is geologically and oceanographically distinct from the Mariana Trench and is a suitable environment for the comparative study of the hadal biosphere. This trench, with a maximum depth of approximately 8000 m, is located in the eutrophic region of the Northwestern Pacific and is close to Honshu Island, Japan, whereas the Mariana Trench is located under the oligotrophic equatorial Pacific, far from continental margins. In March 2011, the Tohoku Earthquake (M9) occurred at the Japan Trench, thus providing an opportunity to investigate the geochemical and biological effects of disturbed seafloor in coastal to hadal waters and seafloor environments (Kawagucci et al., 2012; Noguchi et al., 2012; Arai et al., 2013; Oguri et al., 2013; Kitahashi et al., 2014; Sano et al., 2014; Nomaki et al., 2016). During the post-earthquake investigations of the Japan Trench, it became evident that some of the abyssal and bathyal waters were highly affected by the suspended sediments at 36 days after the M9 earthquake: the microbial communities in these turbid waters were characterized by a greater abundance of microbial cells and greater enrichments of heterotrophic bacterial components compared to the abyssal and bathyal waters without the influence of suspended sediments (Kawagucci et al., 2012). Indeed, this finding has provided important insight into predicting the driving forces for the formation and development of hadal microbial communities in the Mariana Trench, as described in a later study (Nunoura et al., 2015). However, previous post-earthquake investigations in the Japan Trench region did not cover microbial communities associated with hadal waters, and the effects of suspended sediments on the planktonic microbial community were addressed by using only small subunit (SSU) rRNA gene sequencing analysis (Kawagucci et al., 2012).

Therefore, in this study, we attempted to characterize the spatial distribution of planktonic microbial communities associated with surface to hadal waters in the eutrophic Japan Trench to confirm the geochemical gradients across the different depths. Especially, we examined the potential niche separation of nitrifiers’ communities to understand the effects of organic matter originating from landslides and/or turbulent sediments and the surface primary production on the deep-water microbial communities. The NH_4_^+^ flux serves as an excellent biogeochemical tracer of the decomposition of nitrogenous organic matter supplied from sediments and surface primary production, and the niche separation of nitrifiers may be tightly associated with the availability of electron donors (Blackburne et al., 2007; Martens-Habbena et al., 2009; Sintes et al., 2013).

## Materials and Methods

### Sample Collection

The Japan Trench is located along northeastern Japan and at the northwestern margin of the Pacific Plate. The seawater samples used in this study were obtained by vertical hydrocasts of the CTD-CMS (Conductivity Temperature Depth profiler with a Carousel Multiple Sampling system) during a total of three cruises in 2011 after the M9.0 earthquake, and the sampling stations and water depths are summarized in Supplementary Table S1 and Supplementary Figure S1. Hydrocasts at stations N1, N2, N3, R, and JKEO were conducted in April during the R/V Mirai MR11-03 cruise (JAMSTEC; Kawagucci et al., 2012). Hydrocasts at stations E, F, and G were conducted during the YK11-E03 cruise (JAMSTEC) of in May and those at stations 2, 3, 4, 5, and 6 were conducted during the YK11-E04 cruise in June were carried out by using R/V Yokosuka.

The wired CTD-CMS system of the MR11-03 cruise included a CTD (SBE9 Plus, Sea-Bird Electronics), a CMS (SBE32, Sea-Bird Electronics), 36 Niskin-X bottles (12-liter type, General Oceanics), and a light transmissometer (C-star 25-cm light-path type, WET Lab). The Light Transmission Anomaly (LTA), calculated from the difference between the *in situ* light transmission value (Tr: %) and the value of the transparent layer at intermediate depth for each hydrocast, is used to describe deep-sea water turbidity (Kawagucci et al., 2012). The CTD-CMS system of the YK11-E03 and YK11-E04 cruises consisted of a CTD (SBE11 Plus, Sea-Bird Electronics), a CMS (SBE32, Sea-Bird Electronics), and 12 Niskin-X bottles (12-liter type, General Oceanics). A turbidity sensor (ATU6W-CMP, JFE Advantech) was applied to some of the casts during the YK11-E03 and YK11-E04 cruises.

Seawater samples taken by the Niskin bottles and were sub-sampled immediately after recovery onboard for geochemical and microbiological analyses. The concentrations of DO, NO_3_^-^, NO_2_^-^, Si, and PO_4_^-^ as analyzed on board in the case of the MR11-03 cruise were obtained from the Data Research System for Whole Cruise Information in JAMSTEC (DARWIN)^1^. The seawater samples obtained during the YK11-E03 and YK11-E04 cruises were filtrated by a Millex filter unit with 0.22 μm pore size (Millipore) and stored at -20°C for geochemical analyses. The concentrations of NO_3_^-^, NO_2_^-^, and PO_4_^-^ were then analyzed spectrophotometrically using an automated QuAAtro 2-HR analyzer (BL TEC, Japan) at an onshore laboratory in the YK11-E03 and YK11-E04 cruises.

Samples for cell counting were fixed with neutralized formalin (final conc. 3.7% formaldehyde) and stored at -80°C. Samples for virus counting were fixed with glutaraldehyde (final conc. 1%), frozen by liquid nitrogen and stored at -80°C. For molecular analyses, microbial cells in 2–3 L of each sample were filtered on 47 mm cellulose acetate filter (0.22 μm pore size, Advantech) and stored at -80°C.

### Cell and Viral Particle Counting

For cell counting, each sample was filtered onto a 0.2-μm-pore-size Isopore membrane filter (Millipore, Bedford, MA, USA), after being stained with 4′,6-diamidino-2-phenylindole (DAPI). The cells on these filters were counted with an Olympus BX51 fluorescence microscope (Olympus, Tokyo, Japan) at a magnification of ×1,500. At least 400 cells per sample were counted in more than 20 randomly chosen fields in triplicate. Viral particle counts were performed with a FACSCalibur flow cytometer (Becton Dickinson, Franklin Lakes, NJ, USA) as described previously (Brussaard, 2009). Briefly, each sample was stained with SYBR Green I (at a final concentration of 0.5 × 10^-4^ of the commercial stock; Thermo Fisher Scientific, Waltham, MA, USA) for 10 min at 80°C. Yellow-green fluorescent microspheres with a diameter of 1.0 μm (FluoSpheres; Thermo Fisher Scientific) were used as internal standard. Viral particles were discriminated on plots of side scatter versus green fluorescence.

### DNA Extraction and Amplification

Environmental DNA was extracted from the cells on a cellulose acetate membrane filter using a Soil DNA Isolation Kit (Mo-Bio Lab, Carlsbad, CA, USA) with minor modification. Some of the environmental DNA was amplified using a REPLI-g Mini Kit (Qiagen Inc., Valencia, CA, USA) for molecular analyses as described below. Amplified DNA assemblages were digested by S1 nuclease (Invitrogen) prior to the following studies.

### Molecular Analyses of the SSU rRNA and Archaeal *amoA* Genes

Prokaryotic SSU rRNA gene fragments were amplified with a primer set of 530F and 907R (Nunoura et al., 2012) from the original environmental DNA assemblages using LA Taq polymerase with GC buffer (Takara Bio, Otsu, Japan) as described previously. For archaeal *amoA* clone analysis, gene fragments were obtained with a primer set of Arch-amoAF/Arch-amoAR (Francis et al., 2005) using LA Taq polymerase (Takara Bio) from the amplified environmental DNA assemblages. The amplification conditions and primer sequences for each PCR amplification are summarized in Supplementary Table S2.

In the clone analyses, the amplified DNA fragments were cloned into the pCR2.1 vector (Invitrogen) and the clone libraries were constructed. The inserts were directly sequenced with the M13M4 primer using a genetic analyzer ABI3730xl with Big Dye ver. 3.1. The SSU rRNA and archaeal *amoA* gene sequences with >97% identity were assigned as the same clone type (phylotype) using Sequencher v 5 (Gene Codes, Ann Arbor, MI, USA) and GENETYX-MAC ver. 15 (GENETYX, Tokyo, Japan). Potential chimera sequences were manually detected using GENETYX-MAC ver. 15 and blast analysis and were excluded from the further analyses. Also excluded were sequences that were closely related to the potential contaminants, which belong to the genera that are typically found in soil and the human body and that are often detected in the previous negative control experiments of PCR in our lab, such as *Bradyrhizobium, Brevundimonas, Burkholderiaceae, Delftia, Erythrobacter, Lactococcus, Legionella, Methylobacterium, Mycobacterium, Neisseria, Novosphingobium, Propionibacterium, Sphingobium, Sphingomonas, Sphingopyxis, Staphylococcus, Stenotrophomonas*, and *Streptococcus*.

After omitting potential chimeric sequences and potential experimental contamination sequences, all of the SSU rRNA gene sequences obtained in this study were compared using the UniFrac program (Lozupone et al., 2006). An alignment of each SSU rRNA gene clone library was constructed using the SINA alignment service^2^ (Pruesse et al., 2012). A phylogenetic tree of the SSU rRNA gene sequences obtained in this study was constructed by the neighbor-joining method using Clustal X ver. 2.0 (Larkin et al., 2007), and a principal component analysis (PCA) was carried out with UniFrac. Representative SSU rRNA gene sequences were aligned and phylogenetically classified into certain taxonomic units using ARB (Yilmaz et al., 2014). A phylogenetic tree of the thaumarchaeal SSU rRNA gene was constructed with Clustal X based on the unambiguous nucleotide positions. Representative *amoA* sequences were automatically aligned with closely related nucleotide sequences, and then, a phylogenetic tree was constructed by using Clustal X.

### Quantitative PCR Analyses

The primers, probes and components of the standard mixture used for the quantitative PCR analyses are summarized in Supplementary Table S2. The abundance of each gene was quantified as an average of the duplicate or triplicate analyses. A 7500 Real Time PCR System (Applied Biosystems) was used for the all of the assays. The original DNA assemblages were only used for the quantification of the prokaryotic and archaeal SSU rRNA genes, and the amplified DNA assemblages described above were used for the quantification of other genes. The abundance of nitrifier genes in each water sample was estimated from the relative abundance of the archaeal SSU rRNA gene and the respective gene in the amplified DNA assemblages.

The quantification of the archaeal and prokaryotic SSU rRNA genes was performed using primer and probe sets described previously (Takai and Horikoshi, 2000) with minor modifications as described below. Premix Ex Taq^TM^ (Perfect Real Time; Takara Bio) was used for the master mix solution, and a probe was added with a final concentration of 0.2 pmol/μl in PCR reaction solution. The SSU rRNA gene mixtures for obtaining standard curves for each experiment were prepared as follows: a mixture of *Haloarcula japonica* JCM7785, *Sulfurisphaera* sp. (Takai and Horikoshi, 2000), *Thermococcus* sp., “*Ca*. Caldiarchaeum subterraneum” (Nunoura et al., 2011), and MGI α subgroup pMC1A11 (Takai and Horikoshi, 1999) for the archaeal SSU rRNA gene and that of *Haloarcula japonica, Thermococcus* sp., MGI α subgroup pMC1A11, *Shewanella violacea* JCM10179 and *Alkaliphilus transvaalensis* SAGM1^T^ for the prokaryotic SSU rRNA gene. The detection and quantification of nitrifiers were assessed using the amplified environmental DNA assemblages.

Detections of the alpha- and betaproteobacterial *nxrA* and alpha- and gammaproteobacterial *amoA* were performed using Ex Taq polymerase (Takara Bio) with an Mg^2+^ buffer as described previously (Purkhold et al., 2000; Wertz et al., 2008; Nunoura et al., 2013) (Supplementary Table S2). The abundance of the *Nitrospina* and *Nitrospira* SSU rRNA genes was also examined according to the methods described previously (Graham et al., 2007; Nunoura et al., 2013). To identify the group-specific distribution of each archaeal *amoA* gene group, we modified primer sets used in our previous study (Nunoura et al., 2015) and constructed novel primer sets based on the archaeal *amoA* gene sequences obtained in this study as follows. The nucleotide sequence alignments of the archaeal *amoA* gene were constructed with Clustal X ver. 2.0, and we designed primers that specifically matched the *amoA* sequences in the individual groups A, Ba, Bb, and C/D (Supplementary Table S2). For the preparation of quantitative PCR mixtures for specific SSU rRNA gene and functional genes, we applied qPCR Quick GoldStar Mastermix Plus (Eurogentec, Seraing, Belgium) for the SSU rRNA genes of *Nitrospira* and SYBR Premix Ex Taq II (Takara Bio) for the *amoA* genes and the *Nitrospina* SSU rRNA genes (Stephen et al., 1999; Nunoura et al., 2013, 2015). The amplified products from quantitative PCR using SYBR Premix reagent were confirmed by agarose gel electrophoresis. Especially in the archaeal *amoA* genes, amplification specificity was confirmed by clone analysis for the amplicons from several sample depths.

### Accession Numbers

The SSU rRNA and archaeal *amoA* gene sequences obtained in this study were deposited in the DDBJ/EMBL/GenBank database under the following accession numbers; LC087228–LC087916.

## Results

### Environmental Conditions

Conductivity Temperature Depth casts were conducted for a total of 10 stations above the Japan Trench and one station on the abyssal plain in April, May, and June 2011, which corresponded to 36–46, 75–76, and 95–96 days after the Tohoku Earthquake, respectively (Supplementary Table S1; Supplementary Figure S1). Seasonal differences in the temperature and salinity profiles were observed in shallow waters but were not found in waters deeper than 300 m below sea surface (mbs; Supplementary Figure S2). The temperature and salinity profiles between 100 and 600 mbs in the Japan Trench were disturbed because of the mixing of cold and warm currents (Kouketsu et al., 2005), whereas those at a JKEO abyssal plain station (Cronin et al., 2008) were relatively smooth (Supplementary Figure S2). The temperature and salinity below the bathypelagic zone were very similar to each other, and neither geomorphology nor deep-sea currents seemed to affect the depth profiles of temperature and salinity.

Light transmission anomalies in deep waters, which can be a signature of the turbulent diffusion of sediments associated with earthquake-induced seafloor deformation, were determined in the CTD casts during the MR11-03 cruise in April (Kawagucci et al., 2012; Noguchi et al., 2012). During the cruises YK11-E03 and E04, turbidity instead of light transmission anomalies was determined, except for the CTD casts at trench axis station E (Supplementary Figure S3) because the water depth of trench axis station E exceeded the pressure limit of the turbidity sensor. At all of the stations, including station JKEO, increased turbidity was observed just above the seafloor. The higher turbidity in shallow waters was likely due to the greater biomass associated with the vigorous primary production in this region, and the peaks of turbidity in abyssal and bathyal waters at some of the stations may have reflected flows of turbulent sediments associated with earthquake-induced seafloor deformation (Kawagucci et al., 2012).

No apparent anomaly was observed in the profiles of the DO and nutrient concentrations (Supplementary Figure S4). Nitrate at the sea surface was depleted, and nitrate concentrations greater than 40 μmol/kg were observed from 600 to 2000 mbs. The nitrate concentration in bathyal and hadal waters was constant within a range of 35 to 37 μmol/kg. The nitrite concentrations at station JKEO were greater than 0.2 μmol/kg in shallow waters above 150 mbs, whereas nitrite at the trench axis station E was almost depleted (<0.1 μmol/kg) in shallow water except for at a depth of 200 mbs. Nitrite was depleted below 200 mbs at all of the stations.

### Microbial and Viral Abundance

**FIGURE 1:**
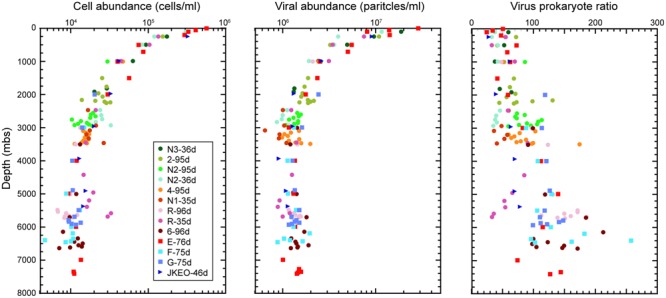
**Microbial cell and viral abundance and virus-prokaryote ratio throughout the water columns in the Japan Trench region.** Each color indicates the sampling stations and the sampling time (days) after the Tohoku Earthquake on March 11, 2011.

The abundances of microbial cells and viral particles (large virus-like particles that can be detected by flow cytometry) in the water samples was evaluated by direct cell counting under a fluorescence microscope and by viral counting using flow cytometry, respectively (**Figure 1**). An overall trend was that the abundance of microbial cells and viral particles decreased with increasing depth to approximately 3000 mbs and was relatively constant below this depth. The maximum cell and viral abundance was 5.7 × 10^5^ cells/ml and 2.9 × 10^7^ particles/ml in the surface water at station E. Virus prokaryote ratio (VPR) values above 2000 mbs were relatively constant, and most of them ranged from 20 to 60, but the VPR values below 2000 mbs were higher and more variable than those in the shallower waters above 2000 mbs and ranged from 33 to 256. Variable patterns in cell and viral abundance and VPR value were observed in many of the water samples close to the seafloor. In particular, the cell abundances in the bathypelagic waters at station N2 and the abyssal waters at station R collected 36 days after the earthquake, was higher than that at stations 3 and 5, respectively, obtained 96 days after the earthquake (Kawagucci et al., 2012); the viral abundances exhibited the opposite trend.

**FIGURE 2:**
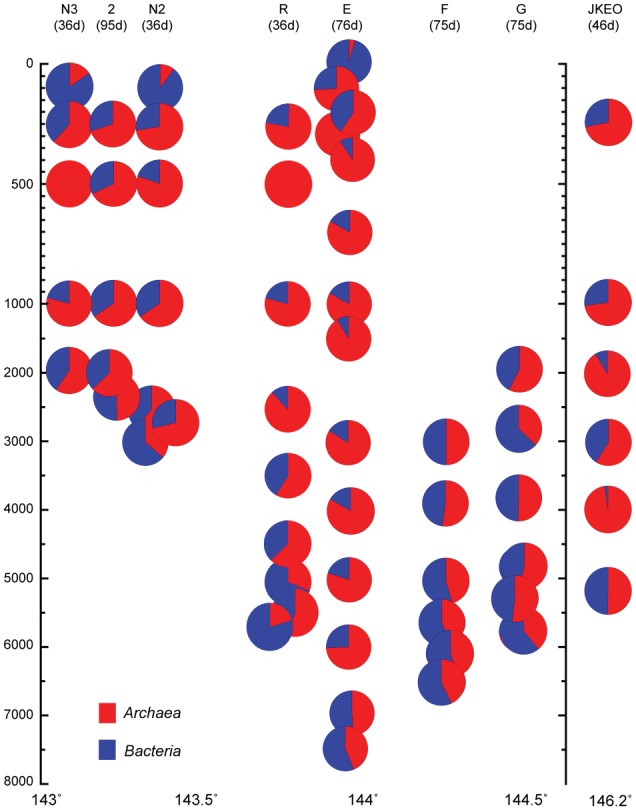
**Relative abundance of the archaeal SSU rRNA gene in the whole prokaryotic SSU rRNA gene assemblage, as estimated by quantitative PCR of the SSU rRNA gene throughout the water column in the Japan Trench region.** The sampling time (days) after the Tohoku Earthquake on 11 March, 2011, is shown in the parentheses. Water samples from the deepest depth for each station were taken above 10 m from the seafloor.

The abundance of prokaryotic communities was also evaluated by quantitative PCR analysis for prokaryotic and archaeal SSU rRNA genes in samples from a total of eight stations (**Figure 2**; Supplementary Table S3). The relative abundance of the archaeal SSU rRNA gene in the entire prokaryotic SSU rRNA gene assemblage was very low in surface waters (4%), and the archaeal SSU rRNA gene populations dominated in the entire prokaryotic SSU rRNA gene assemblages in deep-water zones (**Figure 2**). However, at depths close to the seafloor, the relative abundance of the archaeal SSU rRNA gene in the entire prokaryotic SSU rRNA gene assemblage decreased (**Figure 2**). In addition, the bathyal to abyssal waters at stations F and G, which had higher turbidity, showed relatively low archaeal rRNA gene populations compared to those of abyssal and bathyal waters at trench axis station E. In all of the stations, the highest relative abundance of the archaeal SSU rRNA gene was found in the mesopelagic waters.

### Distribution and Composition of SSU rRNA Gene Communities

**FIGURE 3:**
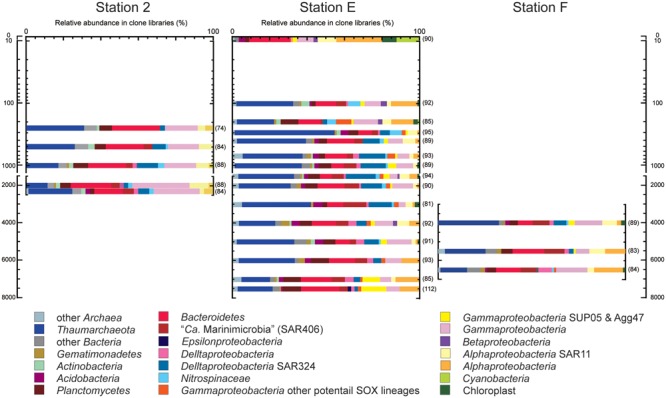
**Prokaryotic SSU rRNA gene phylotype compositions, as obtained by clone analysis in the waters from trench axis station E and continental and oceanic slope stations 2 and F, respectively, in the Japan Trench.** Seawater samples from stations E, 2, and F were taken 76, 95, and 76 days after the Tohoku Earthquake on March 11, 2011, respectively. The numbers parentheses indicate the numbers of sequences obtained from each clone library.

A prokaryotic SSU rRNA gene clone analysis was applied for environmental DNA assemblages from trench axis site station E (sea surface to hadal waters; 0–7404 mbs), station F on the eastern (ocean-side) slope (abyssal to hadal waters; 3992–6487 mbs) and station 2 on the western (continental-side) slope (mesopelagic to bathyal waters; 249–2250 mbs; **Figure 3**). No amplicons of negative control PCR amplification were observed in the gel electrophoresis, and a few sequences were identified as potential contaminants in the clone analysis. After removing potential contaminants and chimeras, a total of 2046 sequences from 23 libraries were analyzed: 1372 sequences from the trench axis site at station E (each 81–112 sequence from 15 libraries), 418 sequences from the continental slope site at station 2 (each 74–88 sequence from five libraries) and 256 sequences from the ocean slope site at station F (each 82–89 sequence from three libraries; Supplementary Table S4).

Through the water column of the trench axis station E, potential heterotrophic taxa such as *Bacteroi-detes, Planctomycetes*, and heterotrophic groups of *Gammapro-teobacteria*, were detected as the predominant populations in the SSU rRNA gene clone libraries (**Figure 3**). The SSU rRNA gene community composition in the surface water was dominated by the SSU rRNA gene of *Cyanobacteria* and chloroplasts. *Thaumarchaeota* was not detected and “*Candidatus* (*Ca*.) Marinimicrobia” (SAR406) was found as a minor population in the clone library. The SSU rRNA gene community at 302 mbs showed the highest abundance of *Thaumarchaeota* and the absence of heterotrophic lineages of *Gammaproteobacteria*. Alphaproteobacterial sequences other than SAR11 phylotypes dominated in the SSU rRNA gene communities associated with waters above 200 m and at hadal depths; however, these sequences were detected as relatively minor populations in the mesopelagic, bathyal, and abyssal waters. Except in the surface water, the *Thaumarchaeota* phylotypes were predominant populations, whereas the *Cyanobacteria* and chloroplast sequences were minor populations throughout the water column. Moreover, potential chemolithotrophic lineages, such as SAR324 (Chitsaz et al., 2011; Swan et al., 2011; Sheik et al., 2014), *Nitrospina* (Spieck et al., 2014), and sulfur-oxidizing groups of *Gammaproteobacteria* were also detected. The SAR324 phylotypes represented one of the dominant populations in the mesopelagic, abyssal, and bathyal waters, but became a minor population in the hadal waters. The *Nitrospinae* phylotypes emerged in the SSU rRNA gene communities below 100 mbs and particularly dominated the SSU rRNA gene communities at depths between 100 and 300 mbs. In addition, the gammaproteobacterial sulfur oxidizers represented by SUP05 and Agg47 (Swan et al., 2011; Marshall and Morris, 2012) were detected throughout the water column, and notably, the relative abundance of SUP05 group increased in the hadal waters. Predominance of heterotrophic *Halomonas* and *Pseudomonas* population found in the waters below 9000 mbs in the Mariana Trench (Nunoura et al., 2015) did not occur in this station.

The relative abundance of archaeal sequences in the clone libraries from trench axis station E ranged from 19 to 55%, except in the library of the surface water (1%). The average abundance of archaeal SSU rRNA genes in the entire prokaryotic SSU rRNA gene populations as estimated by quantitative PCR was 43%, except in the surface water. The archaeal abundance profiles that were obtained by the clone and QPCR analyses were similar (*R* = 0.92), although both analyses might be influenced by biases in PCR. Potential ammonia-oxidizing *Thaumarchaeota* phylotypes predominated in most of the archaeal SSU rRNA gene communities. The MGI group α phylotypes represented the predominant population throughout the water column in shallower depths, except for at 300 mbs and in abyssal waters (Supplementary Figures S5 and S6), and MGI group β phylotypes were also the abundant population, except at 202 mbs. Higher abundances of group α than group β phylotypes were found in the shallow waters at 101, 202, and 403 mbs and in hadal waters.

**FIGURE 4:**
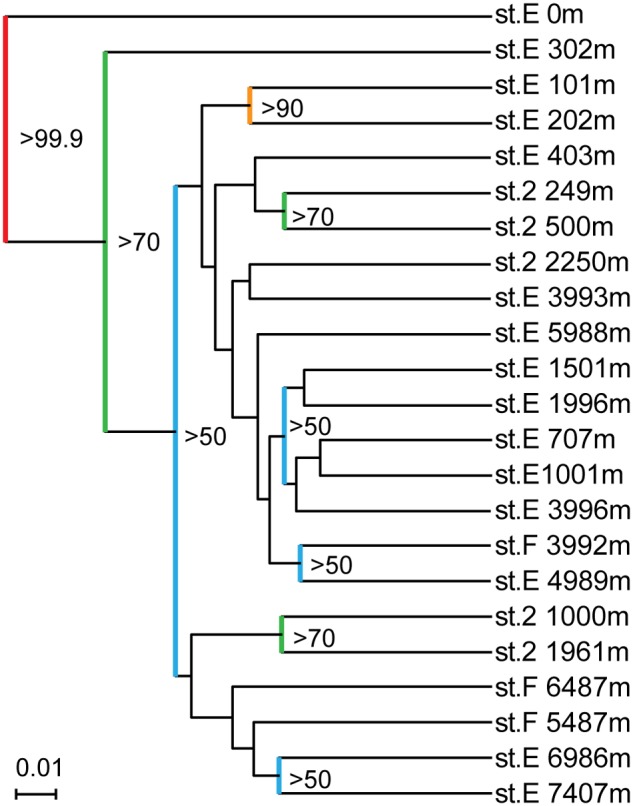
**Result of running Jackknife Environment Clusters of the SSU rRNA gene clone libraries obtained from the waters in the Japan Trench.** Nodes recovered 99.9% of the time are red, 90–99.9% are orange, 70–90% are green, and <50% are blue.

All of the SSU rRNA gene communities obtained in this study were compared using the UniFrac principal coordination analysis (PCA) and Jackknife clustering analysis. In the PCA analysis, the SSU rRNA gene communities from continental slope station 2 shared unique positions compared to those from the other two stations E and F (Supplementary Figure S7). The SSU rRNA gene phylotype compositions from station 2 harbored relatively higher abundances of the potential heterotrophic *Bacteroidetes* and *Gammaproteobacteria* populations than those at the other sites. The analysis also presented a similarity of the SSU rRNA phylotype compositions between the hadal waters of station E (6989 and 7407 mbs) and the abyssal to hadal waters (5487 and 6487 mbs) from station F, whereas those in the abyssal waters (4989 and 5988 mbs) from station E were more similar to those in the bathyal and mesopelagic waters of the same site (1001–3996 m). In the Jackknife clustering, two distinct branches and a major cluster were identified (**Figure 4**). The two distinct branches were the SSU rRNA gene communities in the surface water and the shallow water of 300 m at station E. In the major cluster, a distinctive subcluster constituted each water mass of the lower photic zone (101 and 202 mbs), the upper mesopelagic zone (250–500 mbs), the lower mesopelagic, bathyal, and abyssal zones (700–6000 mbs), and the hadal zone (6986 and 7407 mbs).

### Detection and Quantification of Nitrifiers’ Populations

To clarify the distribution and niche separation of potential ammonia oxidizers that respond to the availability/flux of ammonia/ammonium, the archaeal *amoA* gene community was investigated for the water column at trench axis station E by clone analysis, and subsequent quantitative PCR analyses for nitrifiers were conducted for a total of eight stations. In these analyses, the amplified environmental DNA was prepared as in the case of a previous study (Nunoura et al., 2015).

The archaeal *amoA* gene amplicons were successfully obtained from all of the samples, including surface water, and the clone analysis revealed the potential niche separation of AOA (Supplementary Figures S8 and S9; Supplementary Table S5). Group A *amoA* gene phylotypes dominated in the shallow waters from the sea surface to 1000 mbs. Group Bb and Ba phylotypes were detected as predominant populations in waters from 100 to 5000 mbs and below 200 mbs, respectively. Group C and D phylotypes were detected below 4000 mbs as relatively minor populations compared to the group Ba phylotypes. Groups A, C, and D have previously been recognized as “high ammonia cluster” (HAC) and Groups Ba and Bb were also known as the “low ammonia cluster” (LAC) in the archaeal *amoA* gene classification (Sintes et al., 2013).

**FIGURE 5:**
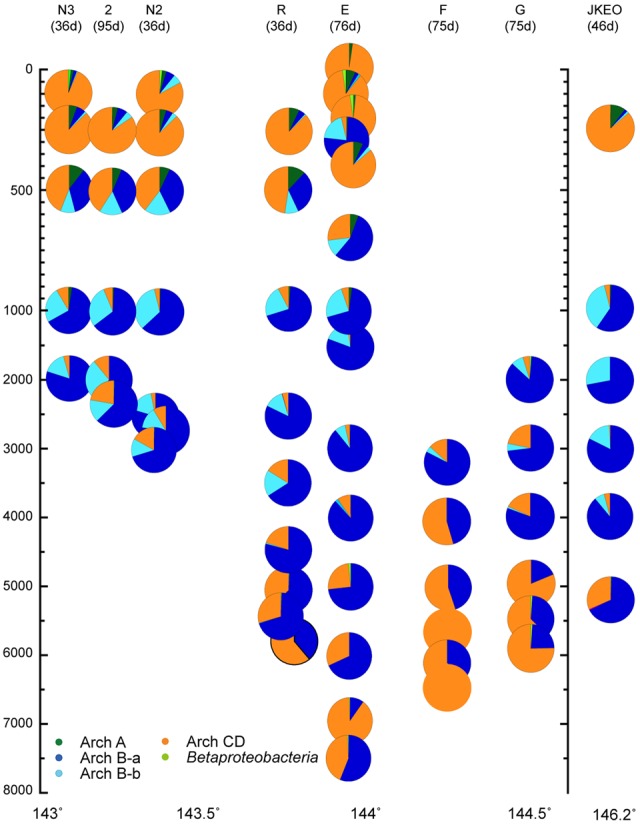
**Relative abundance of the *amoA* gene groups throughout the water column, as estimated by quantitative PCR in the Japan Trench region.** The classification of the archaeal *amoA* subgroups is shown in Supplementary Figure S8. Water samples from the deepest depth for each station were taken at above 10 m from the seafloor. Green, blue, light blue, and orange indicate relative abundance of archaeal *amoA* groups A, Ba, Bb, and C/D, and light green indicate betaproteobacterial *amoA*.

For the quantitative PCR of each archaeal *amoA* group, we modified the primer sequences because several mismatch residues were found between the *amoA* gene sequences found in this study and the group-specific primer sets for archaeal *amoA* gene groups A and Ba that have previously been reported (Nunoura et al., 2015). In addition, a primer set that quantified both groups C and D *amoA* gene phylotypes was newly constructed in this study (Supplementary Table S5). Quantitative PCR for *amoA* genes revealed the niche separation of ammonia oxidizers along the water column (**Figure 5**). Interestingly, no apparent seasonal variation was observed in the distribution pattern of *amoA* associated with shallow waters although the possible seasonal variation was found in the salinity and temperature profiles of the shallow waters. The ammonia-oxidizers community at 302 mbs at station E was distinct from those associated with the shallow waters. The predominant ammonia-oxidizing groups in the waters above 500 mbs were groups C and D, and the predominant group in mesopelagic to bathyal waters was group Ba. The relative abundance of groups C and D increased in the bathyal and hadal waters, and especially in the waters close to the seafloor (**Figure 5**). The greater abundance of group Bb was observed in deeper zones of mesopelagic and abyssal waters (500–3500 mbs; **Figure 5**). Betaproteobacterial *amoA* and group A archaeal *amoA* were found to be minor populations through the water column, whereas group A was detected as a predominant component in the clone analysis of the archaeal *amoA* gene (Supplementary Figure S9). The preferred distribution of group A in relatively shallow waters has previously been reported (Beman et al., 2010; Santoro et al., 2015). In contrast, the relative abundance of betaproteobacterial *amoA* populations increased in the shallow waters and the waters near the sea bottom. The preferential incidence of the betaproteobacterial *amoA* populations in shallow waters was likely consistent with the detection of *Nitrosospira* in waters at 200 and 300 mbs at station E based on the SSU rRNA gene clone analysis. The sum of the archaeal *amoA* copy number in the DNA assemblages below 100 mbs was correlated with the archaeal SSU rRNA gene copy number in a ratio of 0.65 (*R* = 0.91).

In a nitrite-oxidizing bacteria (NOB) population as estimated by quantitative PCR analyses, *Nitrospina* was detected as the predominant group, and *Nitrospira* was found to be a minor population throughout the depths and was absent in some of the samples (Supplementary Figure S10). The relative abundance of *Nitrospira* in the potential NOB population was less than 10% in most of the samples. Relatively higher abundance of *Nitrospira* was found in the shallow waters of station E, and in some of the bathyal and hadal waters. The gammaproteobacterial ammonia oxidizers and alpha- and gammaproteobacterial nitrite oxidizers (e.g., *Nitrobacter* and *Nitrococcus*, respectively) were not detected throughout the water column by conventional PCR amplification.

## Discussion

### Microbial Communities under Eutrophic Ocean

A comparison of the SSU rRNA gene community structures and quantitative PCR analyses for nitrifiers has revealed both similarities and differences in the distribution patterns of microbial communities throughout the water column in the Japan Trench and Mariana Trench (Nunoura et al., 2015). The predominance of potential heterotrophic populations represented by *Bacteroidetes* and “*Ca*. Marinimicrobia” (SAR406) in the SSU rRNA gene communities was observed from the sea surface to hadal waters in the Japan Trench region (**Figure 3**), whereas the potential chemolithotrophic populations; SAR324 and MGI *Thaumarchaeota*, dominate the microbial communities in mesopelagic to abyssal waters in the Mariana Trench (Nunoura et al., 2015). The predominance of group α MGI in the shallow waters above 500 mbs detected was found in the Japan Trench but not in the Mariana Trench (**Figure 5**; Supplementary Figure S6). In the predominant nitrite-oxidizing bacterial populations in hadal waters, *Nitrospina* overcame *Nitrospira* in the Japan Trench (Supplementary Figure S10), whereas the opposite trend has been found in the Challenger Deep (Nunoura et al., 2015). These results indicate that the primary production in the shallow water zone significantly affects the distribution and function of microbial communities, even in much deeper waters. Nevertheless, there are many common features in planktonic microbial communities through the water columns, particularly in hadal microbial communities between the two different oceanographic regions. In both trench regions, it was observed that group γ and group δ MGI predominantly occurred in the lower mesopelagic to abyssal waters and group α MGI in the hadal waters. The potential niche separation of AOA described below suggests the occurrence of higher ammonia/ammonium flux in the hadal waters than that in overlying waters. The lower relative abundance of the potential chemolithotrophic SAR324 population in the hadal waters comparing with the overlying water masses is also found as the common feature of the both trenches. Over all microbial communities in the hadal waters in both trenches would be distinguishable from the communities in the overlying waters (**Figure 4**; Supplementary Figure S7). Accordingly, the potential function of common mechanism(s) and geochemical cycles would lead to the development of similar hadal microbial communities in geologically and oceanographically different trench environments.

### Niche Separation of Nitrifiers

A comparison of the results of the clone and quantitative PCR analyses of the archaeal *amoA* genes confirmed that the archaeal *amoA* gene community structures obtained by the clone analysis were highly biased, as previously reported (Meinhardt et al., 2015; Nunoura et al., 2015). The coordination existed between the thaumarchaeal SSU rRNA and *amoA* gene clusters that were suggested by the quantitative PCR of *amoA* and SSU rRNA gene clone analysis: groups α and C/D, β and A, γ and Ba, and δ and Ba, which is consistent with previous observations (Walker et al., 2010; Swan et al., 2014; Nunoura et al., 2015; Santoro et al., 2015).

It has been pointed out that the niche separation of nitrifier populations is primarily regulated by the availability of electron donors (Blackburne et al., 2007; Martens-Habbena et al., 2009; Sintes et al., 2013). Thus, the niche separation of nitrifiers provides a biogeochemical clue for understanding carbon and nitrogen cycles in oceanic ecosystems, thus highlighting the possible ammonia/ammonium flux/availability in oceanic ecosystems because the concentration in oceanic waters is usually too low to measure accurately. The niche separation of MGI α, δ, and γ in bathyal, abyssal and hadal waters in Japan Trench regions (**Figure 5**; Supplementary Figure S6) was similar to that found in the Challenger Deep (Nunoura et al., 2015), and the results suggest that the availability of ammonium/ammonia in these deep waters are also similar. However, in contrast to the niche separation of AOA in the deep waters, a difference was found in the dominant NOB populations in the hadal waters of the trenches. The higher abundance of *Nitrospina* than *Nitrospira* suggests that the availability of nitrite for NOB associated with the hadal waters in the Japan Trench (Supplementary Figure S10) was lower than that in the hadal waters of the Challenger Deep, in which *Nitrospira* predominated in NOB (Nunoura et al., 2015).

In addition to the ammonia/ammonium concentration, sunlight, pH, and salinity seem to be significant factors that affect the niche separation of ammonia oxidizers (Mosier and Francis, 2008; Gubry-Rangin et al., 2011; Merbt et al., 2012) in the shallow waters in the Japan Trench region. The salinity variation in this study was quite small (<1.5) and is likely negligible throughout the water columns (**Figure 5**; Supplementary Figure S2). The effect of sunlight might be limited in the surface waters in this oceanic region, given the high turbidity in the shallow waters, and the presence of AOA was confirmed even in the surface water by quantitative PCR. The highest pH value of the water column is generally observed in surface waters, but the group α MGI (group C/D *amoA*) dominated in the AOA communities of both the surface and hadal waters. Thus, only a small impact of pH variation was expected in the niche separation of ammonia oxidizers in the oceanic microbial communities.

The predominance of group α (group C/D *amoA* genes) in the shallow waters in the Japan Trench region would be associated with ammonia/ammonium flux and/or sunlight irradiation. In tropical and subtropical oceans, the group β MGI (group A *amoA* genes) predominates in AOA communities in shallow waters (Beman et al., 2008; Santoro et al., 2015). The pH values of the Japan Trench region are slightly higher than those in the surface waters of tropical and subtropical regions including the Mariana Trench region (Pelejero et al., 2010). However, significant differences are expected in the availability of ammonia/ammonium and the extent of sunlight irradiation between the eutrophic Japan Trench region and the oligotrophic tropical or subtropical regions (Sarmiento and Gruber, 2006). In fact, the presence of AOA in surface water in the Japan Trench was detected by quantitative PCR of *amoA* genes in this study but has not been detected in the surface water of the Mariana Trench region (Nunoura et al., 2015). In addition, the transition depth between the predominance of the HAC and LAC groups in the Japan Trench sites is estimated to be 500–700 mbs (**Figure 5**), whereas it has been estimated to be 150–200 mbs in the Mariana Trench (Nunoura et al., 2015). These results suggest that higher primary production and the subsequent higher heterotrophic activity would occur in the surface waters of the Japan Trench region and that the heterotrophic microbial communities would be spread in the deeper water zones of the Japan Trench than in tropical and subtropical oceanic regions. Accordingly, it seems likely that the anomalous abundance of LAC groups (group Ba and Bb *amoA*) in the community of ammonia oxidizers at 302 mbs at station E was likely associated with the anomalous distribution of an oligotrophic water mass from the Kuroshio current, as suggested by the presence of a low-salinity water mass. Considering these factors, pH is not likely to be the primary factor, but sunlight irradiation and/or ammonia/ammonium (and even organic matter) fluxes would significantly affect the niche separation between groups α and β (groups C/D and A *amoA*, respectively).

### Effects of Suspended Sedimentary Organic Matter on Microbial Communities in the Japan Trench

The effects of suspended sedimentary organic matter in the deep waters associated with the earthquakes on the distribution and composition of planktonic microbial communities was confirmed in this study. The importance of suspended organic matter, including both sinking and suspended organic matter, in microbial communities had been indicated only in the bathypelagic waters just above the seafloor (Aristegui et al., 2009; Baltar et al., 2009). In a previous study, the suspended organic matter associated with earthquake-induced seafloor deformation (landslides and turbulent sediments) has been shown to affect the microbial cell abundance and composition of abyssal microbial communities in the Japan Trench region (Kawagucci et al., 2012). In this study, the effect of the suspended organic matter associated with earthquake-induced seafloor deformation on the viral abundance was identified, and the impact on the viral abundance was likely delayed compared to that on microbial abundance (**Figure 1**). In addition, the compositional changes of ammonia oxidizers’ populations and other microbial components responded to the anomalous distribution of high turbidity, especially in the abyssal waters of the ocean-side slope stations (**Figures 3–5**). Moreover, a significant similarity of the SSU rRNA gene phylotype compositions was observed between the hadal waters of the trench axis and the abyssal waters with high turbidity (**Figure 4**; Supplementary Figure S3). These results indicate that the distinctive hadal microbial ecosystems are driven by suspended sedimentary organic matter from the trench slope, as hypothesized in a previous study of the Challenger Deep (Nunoura et al., 2015). Nevertheless, the effect of suspended sedimentary organic matter on the microbial communities is still unclear in the bathyal waters of the continental slope. The effect of suspended sedimentary organic matter on planktonic microbial communities would occur after landslide and turbulent sediment events, although we cannot accurately estimate when landslides and turbulent sediments occur or their duration. The recent landslides and turbulent sediments in the bathyal waters may result in a high turbidity water mass but do not foster planktonic microbial communities that respond to the events. Earthquake-dependent and/or –independent frequent landslides and turbulent sediments and the suspended sedimentary organic matter significantly affects the distribution, composition and function of planktonic microbial communities in trench environments and may lead to the formation and development of a distinctive hadal microbial ecosystem, together with the specific geomorphological and hydrotopographic features of trench valleys.

The methods applied in this study hindered a complete view of the planktonic biosphere in the water columns from sea surface to hadal ocean, especially in the lack of organic geochemistry and functional genomic information. In addition, we cannot excluded the possibility of the PCR biases that might influence the overview of the planktonic biosphere. We will in the future measure concentrations of organic compounds and conduct less biased molecular approaches such as metagenomics and/or transcriptomics to revel the complete view, role and functions of planktonic biosphere in deep oceans.

## Author Contributions

Onboard sample processing and analysis and observatory operations were performed by TN, YY-T, MN, SK, JM, OK, HM, MS, and KT. Cell and viral counts were done by MH and YY-T. Molecular analyses were conducted by TN and MH. Data processing was performed by TN, TY, and YT.

## Conflict of Interest Statement

The authors declare that the research was conducted in the absence of any commercial or financial relationships that could be construed as a potential conflict of interest.

## Footnotes

1 http://www.godac.jamstec.go.jp/darwin/e

2 http://www.arb-silva.de/aligner/
