# Supplementary material for: Distribution and Niche Separation of Planktonic Microbial Communities in the Water Columns from the Surface to the Hadal Waters of the Japan Trench under the Eutrophic Ocean
Source: Front Microbiol. 2016 Aug 10;7:1261. doi: 10.3389/fmicb.2016.01261 (PMC4978738; doi:10.3389/fmicb.2016.01261)
Supplement: Supplementary file 6 [file Image_1.PDF]

Supplementary Information

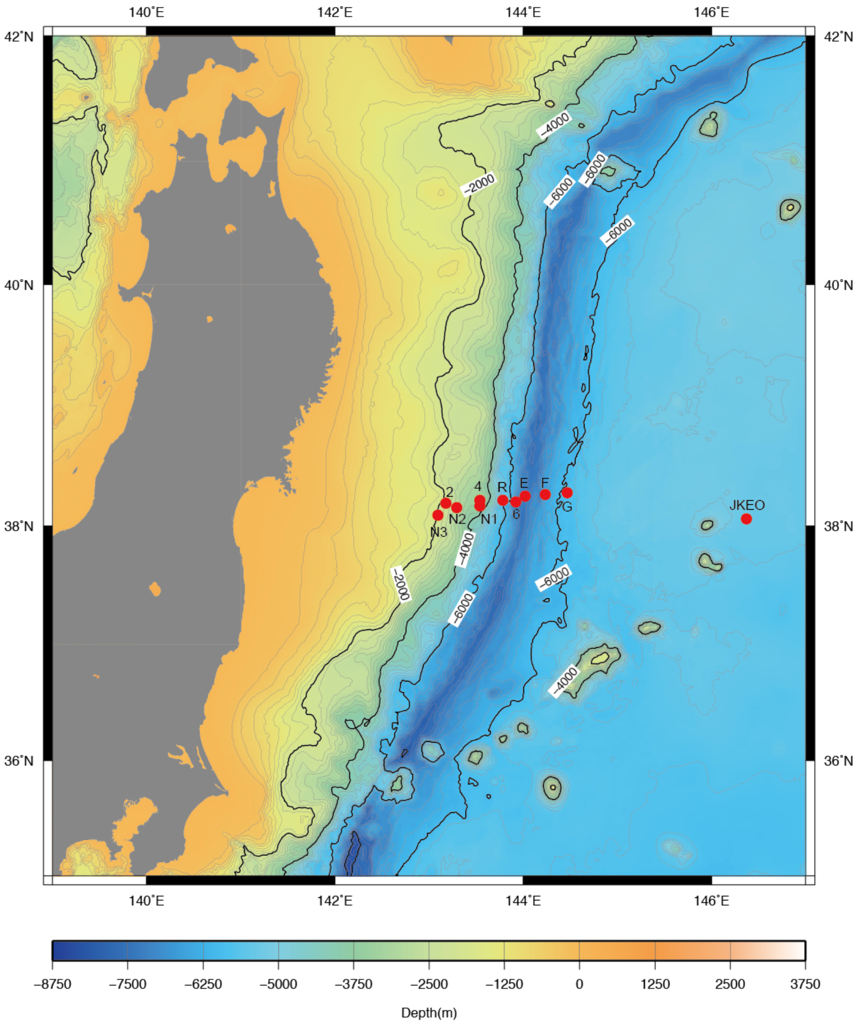

Supplementary Figure S1. Water sampling stations in this study. The bathymetry map was constructed in JAMSTEC using a dataset published by NOAA.

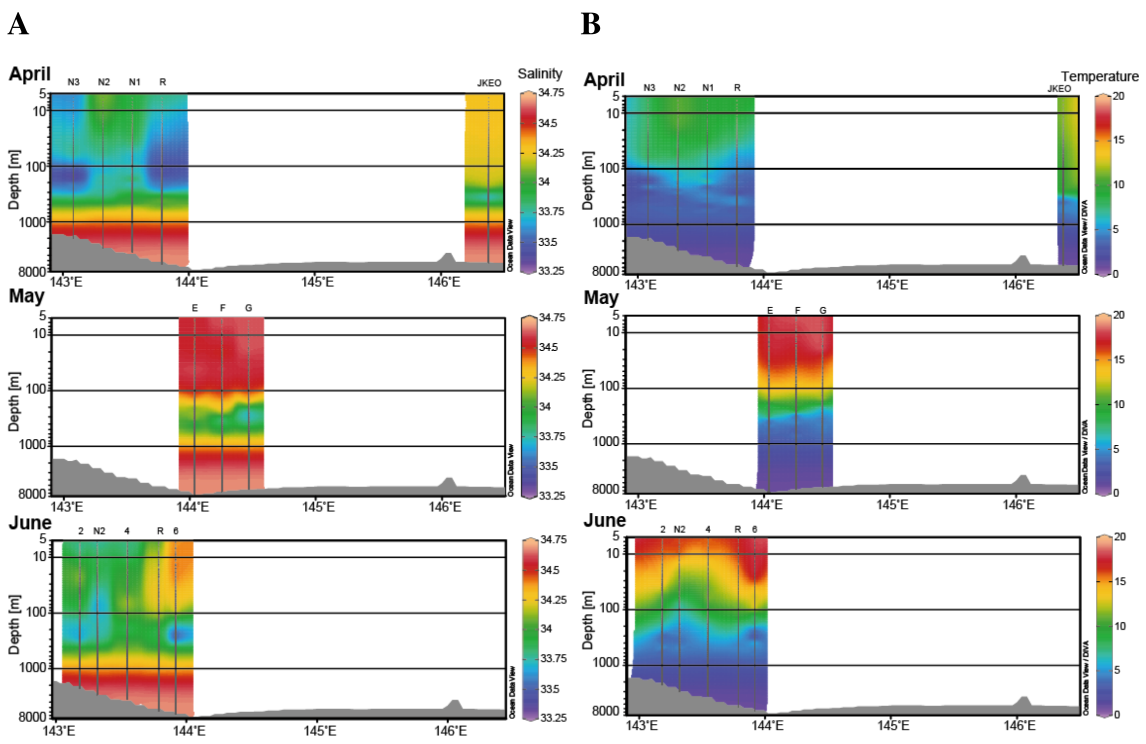

Supplementary Figure S2. Salinity (A) and potential temperature (B) profiles of the water columns in the Japan Trench region in April, May and June 2011. The figures were made by using Ocean Data View (http://odv.awi.de).

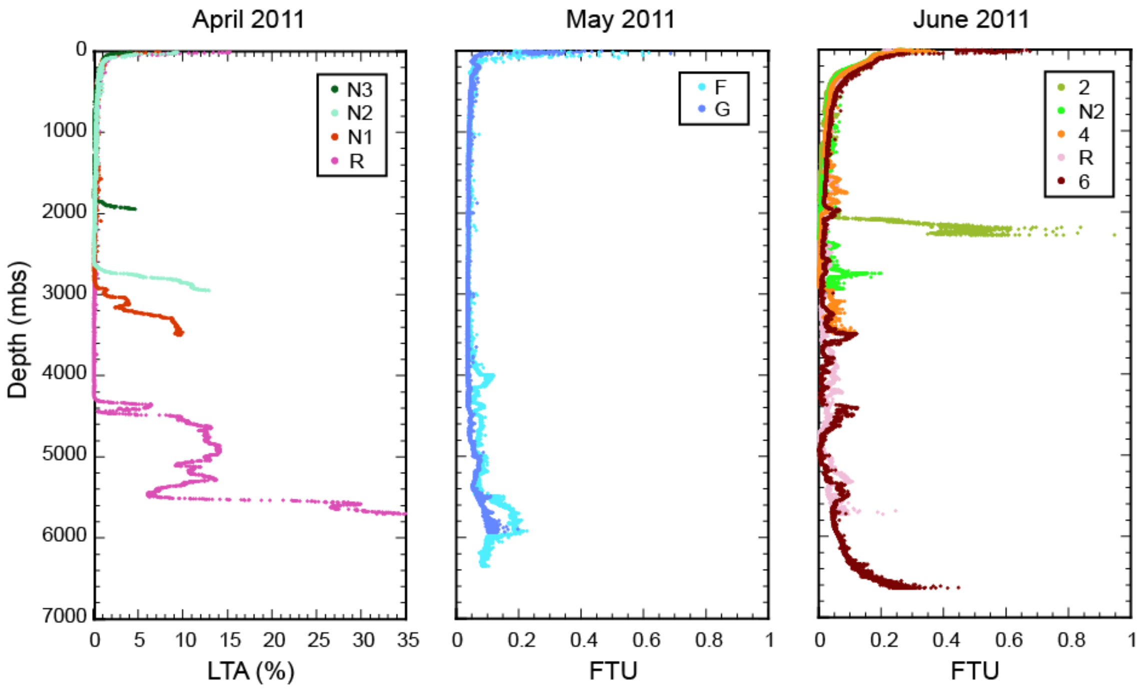

Supplementary Figure S3. Depth profiles of the turbidity of the water columns at stations N3, N2, N1 and R in April; at stations F and G in May; and at stations 2, N2, 4, R and 6 in June. The turbidity of stations N3, N2, N1 and R in April is presented as the Light Transmission Anomaly (LTA) based on the measurements using a light transmissometer (C-star 25-cm light-path type). The turbidity in other stations as measured by a turbidity sensor (ATU6W-CMP) in May and June is presented in Formazin Turbidity Units (FTU). The plots of deepest depth for each station are given at above 10 m from the seafloor.

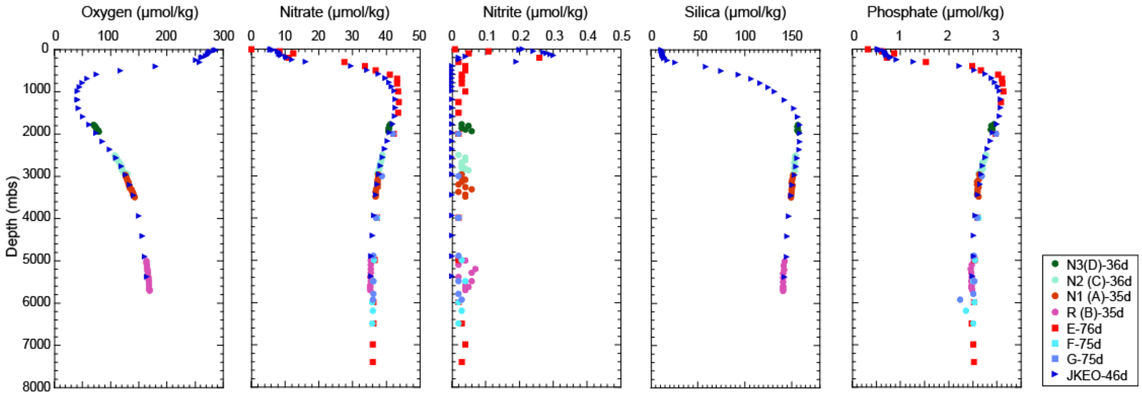

Supplementary Figure S4. Concentrations of dissolved oxygen (DO), nitrate, nitrite, silica and phosphate in the Japan Trench region. Each color indicates the sampling station and times (days) after the Tohoku Earthquake on March 11, 2011.

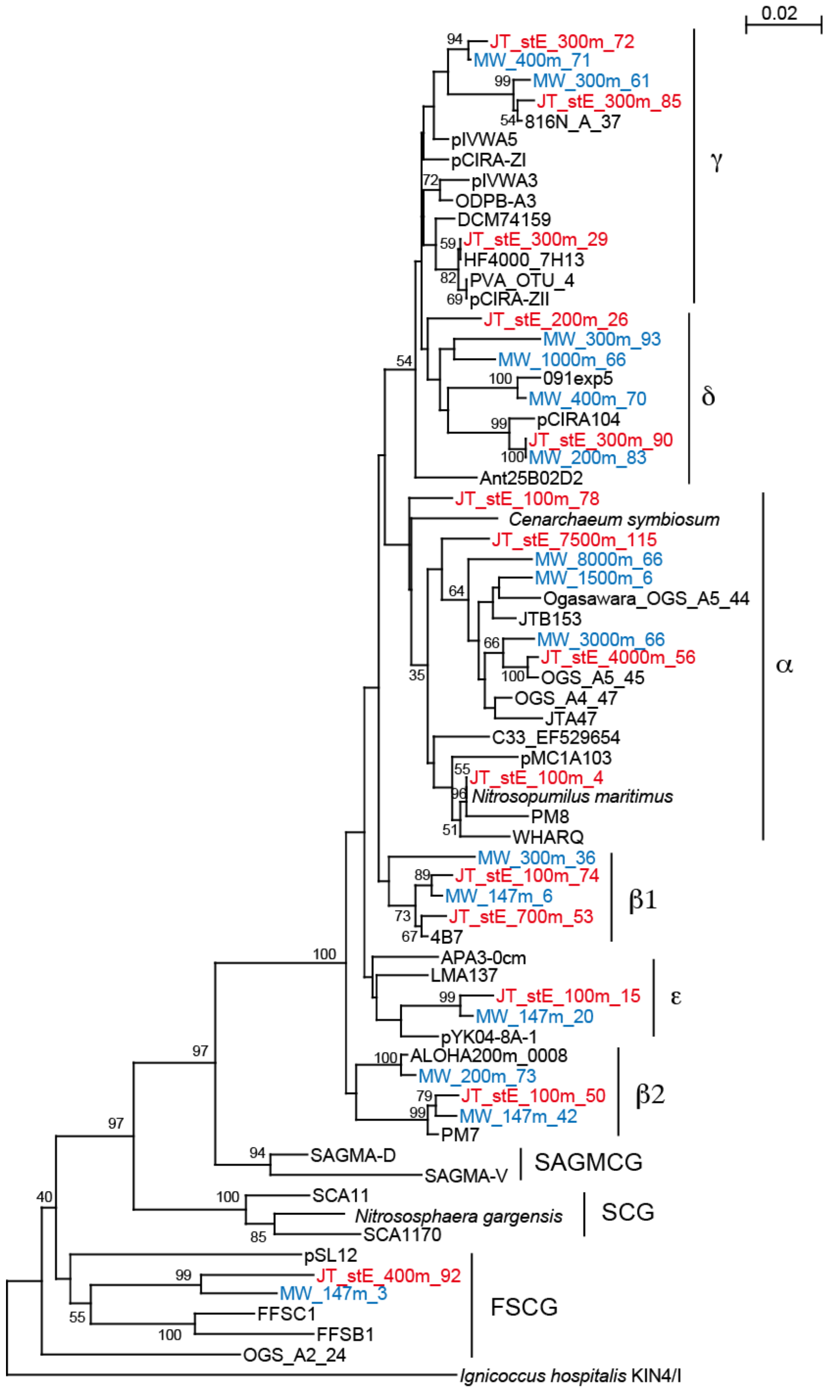

Supplementary Figure S5. A SSU rRNA gene phylogenetic tree of marine *Thaumarchaeota* (MGI archaea) obtained from the water column at station E, constructed by using the neighbor-joining methods for 384 homologous positions. The numbers indicate bootstrap values (>50) obtained from 100 trials. Red and blue characters indicate sequences obtained from the Japan Trench and the Challenger Deep, respectively. The classification of MGI subgroups follows Massana *et al*. (2000) and

Takai *et al*. (2004).

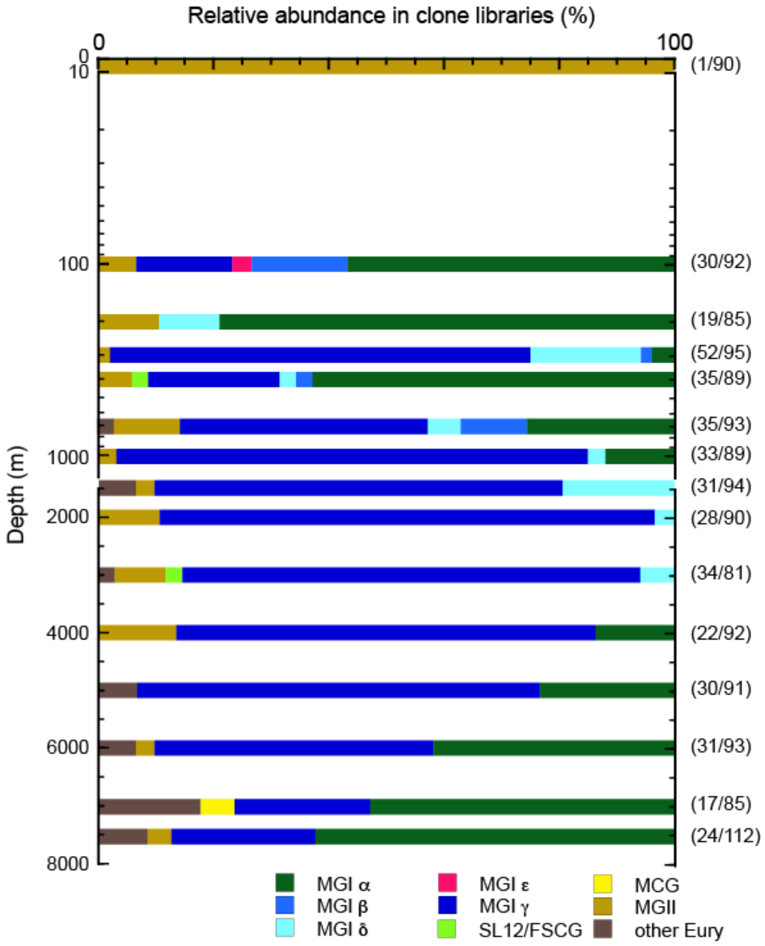

Supplementary Figure S6. Archaeal SSU rRNA gene community structures obtained from the results of the clone analysis of the prokaryotic SSU rRNA gene for the water column at station E. The numbers in parentheses indicate the abundances of thaumarchaeal sequences in the prokaryotic SSU rRNA gene clone libraries. The classification of MGI subgroups is followed by the phylogenetic analysis, as shown in Figs S5.

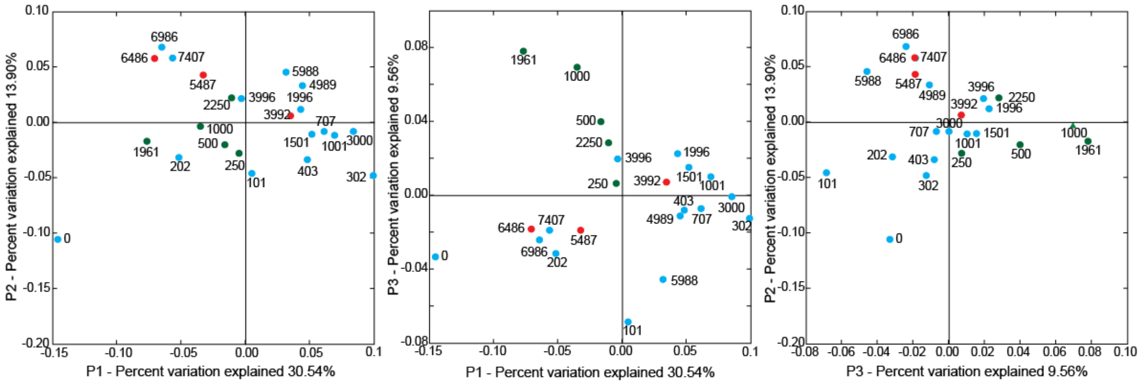

Supplementary Figure S7. PCA analysis of the prokaryotic SSU rRNA gene community compositions in the water samples from stations 2, E, and F. Green, blue and red dots indicate communities from stations 2, E, and F, respectively. The numbers associated with the dots indicate sampling depths.

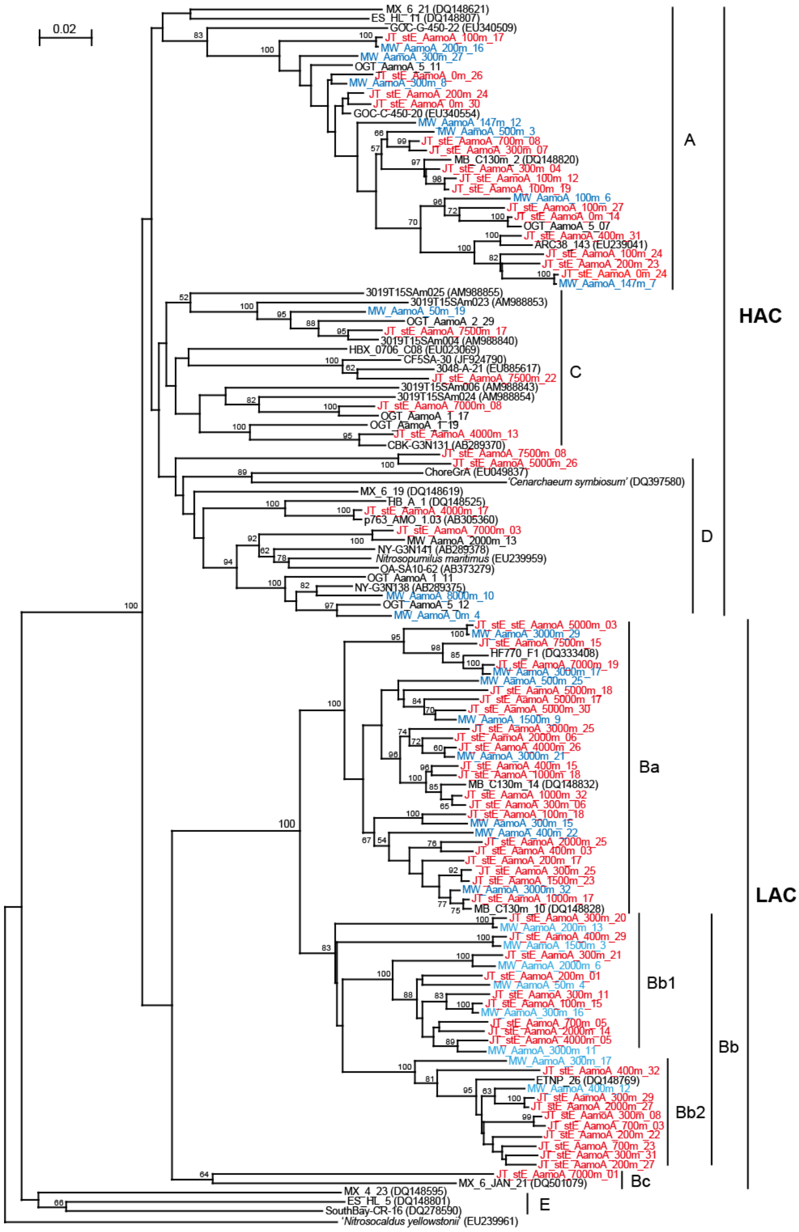

Supplementary Figure S8. Phylogenetic tree of the archaeal *amoA* gene phylotypes obtained from the water column at station E as constructed by using the neighbor-joining method with 588 nucleotide positions. The numbers indicate bootstrap values (>50) obtained by 100 trials. Groups A and B are defined in Beman *et al*. (2008), and C and D are defined in Nunoura *et al*. (2013). The ecotypes of high ammonia concentration (HAC) and low ammonia concentration (LAC) are described in Sintes *et al*. (2013). Red and blue fonts indicate sequences obtained from the Japan Trench and the Challenger Deep, respectively.

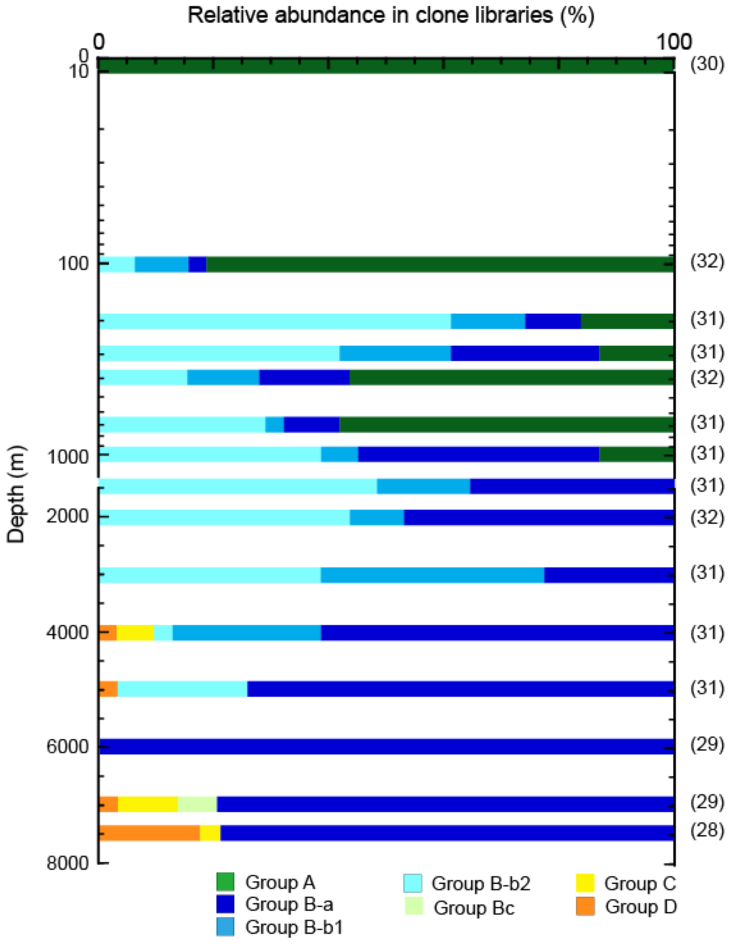

Supplementary Figure S9. Thaumarchaeal *amoA* gene community structures through the water column at station E. The numbers in parentheses indicate the number of sequences obtained. The classification of marine thaumarchaeal *amoA* groups was followed by the phylogenetic analysis shown in Figs S8.

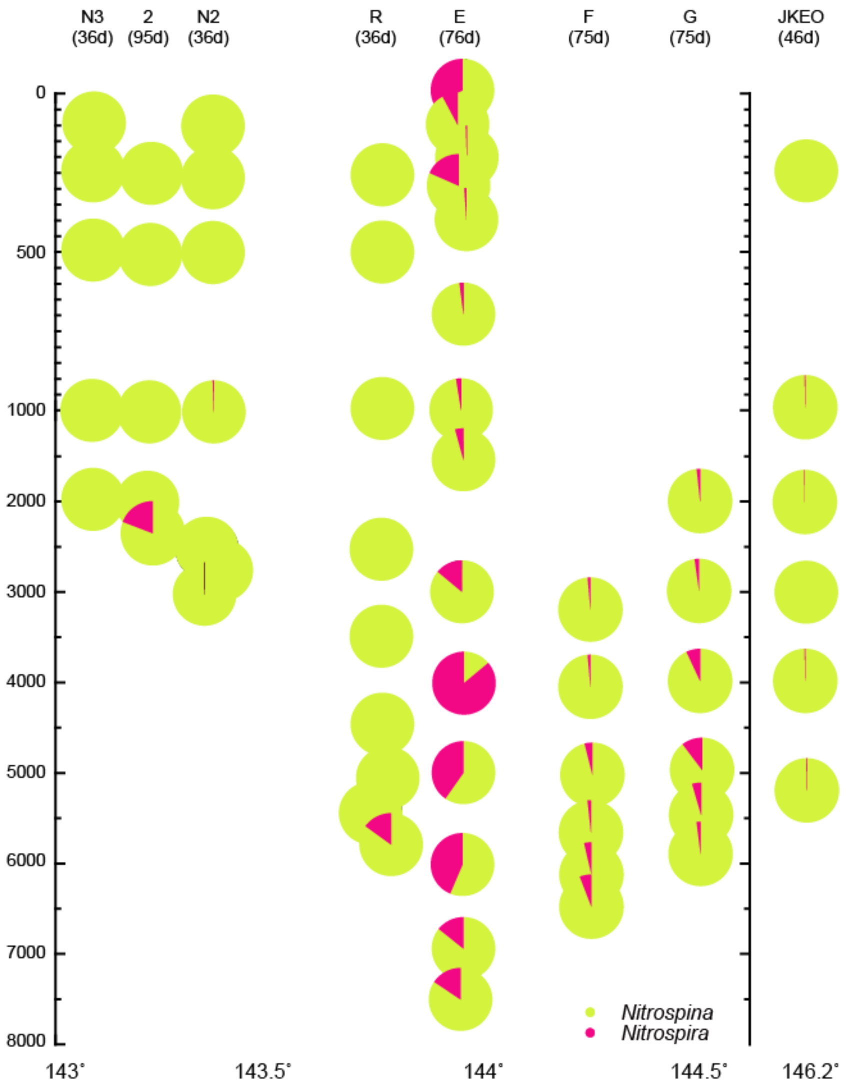

Supplementary Figure S10. Relative abundance of the SSU rRNA genes from *Nitrospina and Nitrospira* throughout the water column estimated by quantitative PCR in the Japan Trench region. Water samples from the deepest depth for each station were taken at above 10 m from the seafloor.

References

Beman, J.M., Pop,p B.N., and Francis, C.A. (2008) Molecular and biogeochemical evidence for ammonia oxidation by marine Crenarchaeota in the Gulf of California. *ISME J* **2:** 429–441.

Massana, R., DeLong, E.F., and Pedrós-Alió, C. (2000) A few cosmopolitan phylotypes dominate planktonic archaeal assemblages in widely different oceanic provinces. *Appl Environ Microbiol* **66:** 1777–1787.

Nunoura, T., Nishizawa, M., Kikuchi, T., Tsubouchi, T., Hirai, M., Koide, O., *et al.* (2013) Molecular biological and isotopic biogeochemical prognoses of the nitrification-driven dynamic microbial nitrogen cycle in hadopelagic sediments. *Environ Microbiol* **15:** 3087-3107.

Sintes, E., Bergauer, K., De Corte, D., Yokokawa, T., and Herndl, G.J. (2013) Archaeal amoA gene diversity points to distinct biogeography of ammonia-oxidizing Crenarchaeota in the ocean. *Environ Microbiol* **15:** 1647–1658.

Takai, K., Oida, H., Suzuki, Y., Hirayama, H., Nakagawa, S., Nunoura, T. *et al.* (2004) Spatial distribution of marine crenarchaeota group I in the vicinity of deep-sea hydrothermal systems. *Appl Environ Microbiol* **70:** 2404–2413.
